# COVID-19 and Rapid Course Adaptations in Saudi Arabia: An Experiential Learning and Recommendations for Online Education

**DOI:** 10.3389/fpsyg.2021.643203

**Published:** 2021-12-23

**Authors:** Basim Sulaiman Alatni, Ismaila Rimi Abubakar, Saad Arslan Iqbal

**Affiliations:** ^1^Department of Landscape Architecture, College of Architecture and Planning, Imam Abdulrahman Bin Faisal University, Dammam, Saudi Arabia; ^2^College of Architecture and Planning, Imam Abdulrahman Bin Faisal University, Dammam, Saudi Arabia

**Keywords:** course adaptations, online education, student satisfaction, emergency remote education, COVID-19, experiential learning, higher education

## Abstract

The COVID-19 Pandemic has severely impacted educational systems around the globe, necessitating rapid modifications to the educational milieu while safeguarding human health and wellbeing. Following the closure of universities in Saudi Arabia, the instructors of all theory courses were mandated to switch from face-to-face course delivery to remote teaching and learning. This research examines the challenges and impacts of the COVID-19 Pandemic on the mode of teaching and learning and the numerous adaptations in the pedagogical framework of the Landscape Architecture program at Imam Abdulrahman Bin Faisal University, Saudi Arabia. It also explores the opportunities the transition to online education presents to faculty and students moving forward. The data were collected using an online questionnaire survey and focus group discussions. Data analyses consisted of descriptive statistics and thematic content analysis. The research finds that the sudden transition to online teaching and learning disrupted academic activities and had negatively affected the existing teaching and learning framework. Therefore, the research recommends an adaptable and dynamic teaching framework agile enough to cope with sudden disruptions. It concludes with lessons for future teaching and learning frameworks and amendments for upcoming sessions to deal with similar situations.

## Introduction

The World Health Organization (World Health Organization [WHO], 2020) declared on 11 March 2020 that the COVID-19 has become a *Pandemic* after infection cases reached more than 100,000 worldwide. As the COVID-19 continued to spread at an alarming rate, many educational institutions around the globe were forced to shift to online or remote learning options, which caused some teaching and learning challenges among faculty members and students (Marinoni et al., 2020). The situation indicates that educational institutions should be resilient and swift to make necessary decisions and adaptations to ensure proper teaching and learning in challenging times. In addition, researchers suggest that institutional and infrastructure support are important in ensuring the quality of online education (Jones, 2014; Lederman, 2020).

During the Pandemic, the transition to the online environment has required instructors to modify their teaching practices, including assignments and exam formats (Johnson et al., 2020). Because the conventional techniques for assessing student success are not compatible with online settings, modes of course delivery require modification (Serwatka, 2002). While some researchers such as Herrington et al. (2001); Mayes et al. (2011) stress the importance of group work and collaboration in online learning, Dumford and Miller (2018) posit that students taking online classes might experience less collaborative learning. Likewise, Driscoll et al. (2012) emphasize adjusting teaching strategies and methodologies to conform to the requirements of digital technologies. These adjustments include using information and communication technologies (ICT), remote teaching strategies, and untested methods (Driscoll et al., 2012). The sudden transition from face-to-face teaching to remote or online modes of teaching necessitated many abrupt and untested modifications. Therefore, the outcomes of such adjustments can only be revealed as the courses progress (Iqbal and Tayyab, 2021). The transition has notably resulted in an increased workload for instructors and a challenge to shift existing courses designed for face-to-face to an online format (Kaden, 2020). On this account, it is safe to add that online education adaptation requires adopting different teaching methods, which require testing and putting the needed support in place, including guidance, technical and moral supports (Marinoni et al., 2020). The Pandemic challenged educational institutions and the telecommunication sector to meet the high demand for educational technologies from students and faculty. The extent of satisfaction with online teaching and learning depends upon the preparedness and the infrastructure provided by each academic institution.

In Saudi Arabia, the Ministry of Health officially reported the first COVID-19 case on 02 March 2020 (CNN Arabic, 2020). On 08 March 2020, the Ministry of Education suspended the face-to-face mode of education in all educational institutions in the country, mandating them to switch to online or virtual learning modes (Alarabiya News, 2020). The Ministry further advised universities to develop their strategies of switching to the online mode of education and facilitate meeting the needs of faculty and students. Before the Pandemic, e-learning tools, and online methods of teaching in Saudi Arabia were limited (Alali and Xanthidis, 2014; Rajab, 2018). However, some researchers have speculated extensive use of e-learning systems to help students gain wider access to higher education and overcome cultural barriers (Alali and Xanthidis, 2014; Xanthidis et al., 2016; Aldiab et al., 2017; Abubakar et al., 2020). Also, Rajab (2018) indicates that online education provides a safe learning environment for students and can deliver quality education. However, during the Pandemic, technical and technological issues have been highlighted as some of the most crucial factors to be considered for effective e-learning systems (Almaiah et al., 2020).

Imam Abdulrahman Bin Faisal University is among the Saudi universities that suspended all face-to-face academic activities, including classes, training, and workshops during the Pandemic. As such, the University management ensured that while academic activities continue virtually, the health and wellbeing of the students and staff are safeguarded. Furthermore, the University management swiftly provided technical support and extensive training workshops on using various software and applications such as videoconferencing through *Zoom* and using the *Blackboard* and *QuestionMark* for online quizzes and exams to facilitate learning delivery in all programs. These adaptations seem to be unanimously adopted by universities worldwide to facilitate and ease education delivery and help faculty and students deal with anxiety and possible negative effects of the Pandemic (Johnson et al., 2020; Lederman, 2020).

The present study aims to examine the impact of the COVID-19 Pandemic on traditional teaching and learning practices and the opportunities and challenges of switching to an online-centric delivery mode at the Department of Landscape Architecture at Imam Abdulrahman Bin Faisal University, Saudi Arabia. The authors conducted a questionnaire survey and focus group discussions with students and reported a case study of how they adjusted course delivery plans, such as reformatting the remaining lectures, reducing course load, and modifying assessment criteria. This study shares experiential online teaching and learning from the perspectives of both students and instructors of a theory course after the transition from the traditional to the online mode of teaching and learning. Both students and instructors faced considerable obstacles and had to adapt to the new modalities accordingly. Therefore, the questions this study explores are:

1. How did the students perceive the sudden shift from face-to-face to the online mode of learning?
2. What were the common obstacles faced by the students and teachers alike during the online teaching and learning phase?
3. How can instructors improve the student experiences during the online learning phase?

In Saudi Arabia, few studies reported the experience of university students and faculty during the sudden transition to online education caused by the Pandemic. For example, Almaghaslah and Alsayari (2020) studied the impacts of the Pandemic on teaching, research, and administrative duties among the academic staff of the College of Pharmacy at King Khalid University using an online questionnaire survey. The respondents reported that the transition to online education and the related administrative work was carried out smoothly, but research activities were negatively affected. Nevertheless, the study did not include students who are important stakeholders in teaching and learning. On the other hand, Khalil et al. (2020) conducted a qualitative study to assess the perspectives of Qassim University medical students about the sudden transition to online education during the Pandemic and noted that the participants well-received online classes. However, their study neglected the perspectives of academic staff regarding the prospects and challenges of online learning. Similarly, medical and pharmacy students have different learning environments than landscape architecture students, indicating the need also to explore the experience of the latter. Also, the present study utilized mixed methods to benefit from the strength of both questionnaire survey and focus group discussion (FGD).

The experiential learning of a rapid adaptation to online course delivery reported in the present study can guide faculty members in other universities to learn lessons or validate their findings from the course adaptations they made during the Pandemic. Furthermore, the study may give some positive insights about future academic planning considerations in case the Pandemic forces longer university closures or if a similar situation arises in the future. The next section details the adaptation measures for transition to online course delivery at the Department of Landscape Architecture of the University. Next is Section “Materials and Methods” that describes the research design. Section “Results and Discussions” presents and discusses the study findings, while the last section concludes the paper with some remarks and recommendations.

## Adaptations to Online Teaching in the Landscape Architecture Program

In the following days, after the first case of COVID-19 was reported in the country, all faculty and staff were physically coming to the University for regular office hours. However, as the number of new cases increased, the University management directed all faculty members to transit to remote work completely. Thus, the semester was almost halfway through, with substantial course content yet to be covered at that time. All university courses are offered in a 15-week semester followed by a final examination.

The Theoretical courses in the Program are lecture-based, delivered face-to-face using tools such as whiteboard and multimedia. Class meetings are scheduled weekly, and the students are supposed to be involved in class discussions (individual and group) which also account for their final grade. Furthermore, they also watch two documentary films related to the course objectives and summarize them based on a standard procedure developed by the instructors. Other course assessments typically include at-least two individual report writing assignments and one end-of-the-term group project. The students are only required to conduct site visits for the group project.

Additionally, the students must take two written quizzes and one mid-term examination based on the lectures and assignments related to the course. The course concludes with a written examination that has the highest weightage in terms of the grades assigned. The course learning outcomes are based on the learning domains - knowledge, skills, and competencies - approved by the National Center for Academic Accreditation and Evaluation.

### Modes of Communication Between Students and Instructors

The course instructors were available for consultations and student support during office hours, at least 2 h a week. Students were notified about the office hours of all instructors, along with their email addresses. A student who cannot meet an instructor during their designated office hours may request another convenient time. At least one instructor’s contact number is also shared with the students for communicating *via* text messages, WhatsApp, or audio calls in case of urgency.

The e-learning Management System of the University uses Blackboard as the mainstream platform for providing a wide range of services to the students and instructors, including course guides, lessons, updates, assessments, grades, tools. In addition, the Students Information System (SIS) is used to record students’ attendance, grades, and class schedules. Due to a growing emphasis on e-learning technologies, the University and its associated deanships have provided extensive training and workshops to enhance students’ and instructors’ involvement in the e-learning systems. Even before the Pandemic, instructors have frequently used these systems to share course content with the students. However, it is worth mentioning that instructors were mostly using the Blackboard for announcements, sharing lecture resources such as PDFs or PowerPoint slides, and posting students’ grades. Similarly, students were typically using the Blackboard platform to access information provided by the instructors and submit assignments. Other advanced features of the Blackboard, such as online quizzes or exams and audio/video lectures, were less frequented by the course instructors and students mainly because of inconvenience, time consumption, and lack of experience.

### Adaptations Made by the Course Instructors

The course instructors attended two of the many training sessions offered by the University on the use of QuestionMark. They learned how to create quizzes and exam questions and link them to course learning outcomes, set up the date and time for the quiz/test to be available to the students, access and grade the completed quiz or exams, and assign and publish grades on the *Blackboard*. The course instructors also attended one workshop on using the Zoom application and its various features and settings for delivering online lectures and coordinating and recording discussions with the students. The literature indicates that providing faculty with the required training can enhance their satisfaction and technical and pedagogical skills (Stickney et al., 2019).

**FIGURE 1:**
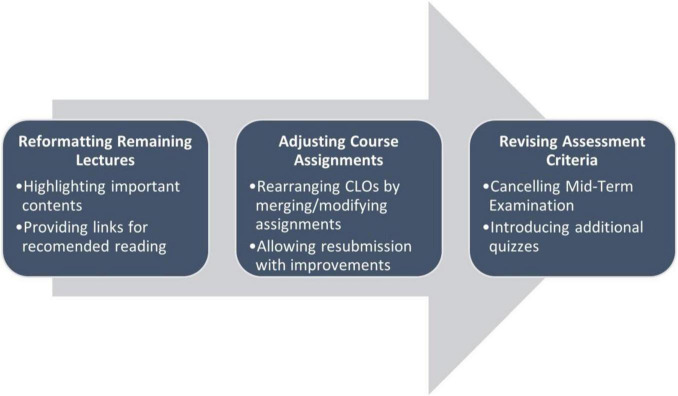
Steps involved before resuming online classes.

The most important step after the closure of the University was to revisit the remaining lectures and assessment tasks and decide whether they require any modifications because of the change from traditional to online mode. Figure 1 below summarizes the major modifications made before resuming the course delivery after the suspension of face-to-face classes.

### Reformatting the Remaining Lectures

Initial discussions among the instructors indicated that the students tended to be less involved and focused on online lectures due to the lengthy online meetings. Therefore, the PowerPoint lectures were slightly modified by either deleting additional text or making the most important words, phrases, or definitions bold or highlighted to help the students understand the contents more easily. However, the students were still given either the extended versions of the lectures for reading at home or a detailed list for ‘recommended reading’ along with hyperlinked sources available through the University’s digital library.

### Adjusting Assessment Criteria and Requirements

During the transition period, the students have already submitted some of their assignments. However, regarding group assignments, the Department decided to merge their course learning outcomes with other remaining assessments because the students could not make site visits due to the lockdown situation. In place of conventional mid-term examination where students appear physically in the exam hall, the instructors revised the assessment by carefully merging grading weightage according to the course learning outcomes. For example, the mid-term and final examinations grades were combined into additional quizzes and individual assignments. This modification shown in Figure 1 ensures that the course learning outcomes and student performance are measured effectively.

The instructors canceled the mid-term examination and introduced three more quizzes instead of only two planned quizzes at appropriate intervals to replace the final exam. Due to the circumstances, the instructors revised the assessment criteria by carefully merging and shuffling grading weightage according to the course learning outcomes. For example, the mid-term and final examinations grades were combined into additional quizzes and individual assignments. This adjustment was necessary because otherwise, the course learning outcomes and student performance would have been affected considerably.

### Enhancing Modes of Communication

To minimize the disruptions caused by the sudden transition to online learning, each course instructor offered two extra office hours a week for one-on-one online consultations with students to address any problems they encountered. Each instructor also maintained several hours responding to student inquiries *via* emails. Instructors also shared their phone numbers with the students for communicating *via* text messages, WhatsApp, or audio calls in case of urgency. In addition, the instructors increasingly used SIS and Blackboard e-learning platforms to record students’ attendance and grades, publish instruction materials and assignments, and interact with students.

### Collecting Regular Feedback From Students

The instructors electronically sent out online snapshot feedbacks in the form of open text comments to each student at the end of each lecture session to collect feedback about students learning outcomes. Additionally, feedbacks on the challenges related to online education were encouraged from the students during the FGD sessions. The discussions were replayed at the end of the sessions, and key issues were transcribed and synthesized. The outcome from the process informs a part of the result section of the present study.

## Materials and Methods

This research adopted a mixed-method approach comprising an online questionnaire survey and FGD to examine the impact of COVID-19 Pandemic on course delivery after the sudden transition from a traditional, face-to-face teaching and learning practice to online education. The study also assesses the opportunities and challenges encountered during the transition, focusing on the course delivery modes, methods of assessment and examinations used by the course instructors, the extent of students’ satisfaction with the course delivery, and the effectiveness of the teaching and learning processes during the initial phase of online education. While the questionnaire survey depended upon the experiences of all students of the Department of Landscape Architecture, the FGD approach was meant to assess students’ satisfaction in an individual course taught by the researchers during the Pandemic. Indeed, FGD can assess students’ experiences of particular teaching and assessment methods (Breen, 2006).

The structured questionnaire was adapted from a previous study (Almaghaslah and Alsayari, 2020) and hosted online. The researchers emailed the link to the survey to all 45 students enrolled in the Bachelor of Landscape Architecture Program at the study time. The questionnaire was structured around the participants’ demographic characteristics, experiences, perceptions about online lecture sessions, assessments, ICT, ability to interact with the e-learning platform, and participation in class discussions. Survey responses were recorded using the Likert Scale (1-5) satisfaction level: never, rarely, sometimes, often, and always. Out of 45 enrolled students, 33 students (73%) completed the survey. The data were analyzed using descriptive statistics: percentage, mean, standard deviation (SD), and the Relative Importance Index (RII).

The FGD was held using the *Zoom* application and lasted for about 50 min. Sixteen out of the 20 enrolled students participated in the discussion sessions, divided into two groups for easier management. The sessions began by briefing the participants about the purpose and importance of the study, the type of questions to be asked, and how they could interact. Next, the course coordinator facilitated the discussion and allocated speaking time to each participant. Then the floor was opened for discussion, comments, and questions and answers. Both sessions were recorded, transcribed, and analyzed using thematic content analysis in which themes from the discussions were generated, organized, and synthesized.

Moreover, this study’s authors, the course instructors, have utilized a single descriptive case study methodology to explain their own experiences before, during, and after shifting to online teaching and learning modes. The approaches used for collecting the case study data include in-class (online) discussions, instructors’ observations, instructors’ daily activity log, feedback through emails, WhatsApp and text messages, phone calls, and notes from the class representative. A study comprising multiple data sources has higher content validity because of triangulation: converging lines of inquiries.

A reliability test using Cronbach alpha was performed on the research variables to test whether the participants’ responses to each research question were valid. The test results in Table 1 indicate a fair coefficient of 0.529 for participants’ ability to interact with peers using the e-learning platform. The rest of the variables have satisfactory reliability, with coefficients ranging from 0.797 to 0.907.

**TABLE 1:** Reliability test of the questionnaire items.

Research variable	No. of items	Cronbach’s α
Ease of technology use	5	0.871
Engagement with online assessment	5	0.812
Degree of concentration during e-learning	5	0.797
Engagement with instructor and peers	12	0.907
Willingness to engage in group and one-to-one discussions with instructors	5	0.853
Ability to interact with peers using e-learning platform	5	0.529

*α ≥ 0.9 = excellent; 0.7 ≤ α < 0.9 = Good; 0.6 ≤ α < 0.7 = acceptable; 0.5 ≤ α < 0.6 = fair; α < 0.5 = unacceptable.*

## Results and Discussion

### Results From the Questionnaire Survey

The survey respondents were drawn from year three to five undergraduate students (*n* = 33) where 42.4% of those that completed the survey were students in their final year (year 5) of the study, 39.4% were from year 4 (4th year), while 18.2% were studying in year 3 (3rd year).

*(a) Students’ experiences with online course delivery and assessments*

Table 2 presents the students’ experience and engagement with online learning using percentages, averages, SD, and relative importance index (RII). There seem to be different levels of challenges and disruptions experienced by the participants. The difficulty of concentrating during classes is the highest-ranked challenge faced by students (RII = 5.34), followed by concentration during lectures with a similar RII of 0.528. In contrast, the least challenge is the internet connection problems (RII = 0.472). While 30.3% of respondents reported always experiencing technical communication problems, 6.1% said they always experienced the challenge. About 12.1% indicated they always face difficulty concentrating on the lecture contents, 24.2% stated they never experienced such a problem. In another question around student ability to keep pace with lecture delivery, 33.3% of respondents sometimes and 15.2% of them often find online lectures a bit fast. Hence, they are unable to keep up with the delivery.

**TABLE 2:** Students’ experience with online lecture delivery.

Variables	Percentage (%)					Mean	*SD*	RII
	Never	Rarely	Sometimes	Often	Always			
Technical problems connecting to e-learning medium for lecture (zoom)	18.2	33.3	36.4	9.1	3.0	2.45	1.003	0.490
Difficulty concentrating during lectures	24.2	24.2	24.2	15.2	12.1	2.67	1.339	0.534
Difficulty understanding the lecture content or concept	27.3	15.2	27.3	27.3	3.0	2.64	1.245	0.528
Difficulty keeping up with lecture pace	18.2	27.3	33.3	15.2	6.1	2.64	1.141	0.528
Experienced technical problems communicating with instructors during live sessions	30.3	12.1	30.3	21.2	6.1	2.36	1.245	0.472

Students were also asked to gauge their level of engagement with varied forms of assessment used to measure their learning outcomes. Table 3 indicates that the most important challenge to the students is feeling of overburden by excessive assignments (RII = 0.63), followed by understanding assignments requirements (RII = 0.57) and completing assignments online (RII = 0.53). On the other hand, the least important challenge is the inability to access assignments online (RII = 0.442). Based on the number of questions asked, 27.3% of the participants said they sometimes have difficulty organizing themselves to prepare for online quizzes/exams at home. While 42.4% of the respondents sometimes experienced difficulties understanding assessment requirements, more than half of the respondents never or rarely faced challenges of understanding or completing online quizzes/exams. Similarly, 66.7% of the participants affirmed that they never or rarely experience difficulties submitting their assessments to Blackboard (Table 3).

**TABLE 3:** Students’ experiences with online assignments and assessments.

Factors	Percentage (%)					Mean	*SD*	RII
	Never	Rarely	Sometimes	Often	Always			
Difficulty in preparing for online quizzes/exams at home	30.3	27.3	27.3	6.1	9.1	2.36	1.245	0.472
Inability to access online quizzes/exams links	24.2	36.4	33.3	6.1	0	2.21	0.893	0.442
Difficulty in concentrating during online quizzes/exams	30.3	36.4	18.2	9.1	6.1	2.24	1.173	0.448
Difficulty in understanding online quizzes/exams	21.2	33.3	27.3	12.1	6.1	2.48	1.149	0.496
Experienced problem completing online quizzes/exams within the given time	24.2	27.3	18.2	21.2	9.1	2.64	1.319	0.528
Problem arranging for facilities and devices required to complete assignment (internet/computer/laptop/printer)	30.3	18.2	24.2	24.2	3.0	2.52	1.253	0.504
Difficulty in concentrating or completing assignments while working from home	27.3	18.2	36.4	15.2	3.0	2.48	1.149	0.496
Understanding the requirement for the assignments and assessment	21.2	6.1	42.4	27.3	3.0	2.85	1.149	0.570
Feeling overburdened due to the excess of other course assignments	9.1	21.2	39.4	6.1	24.2	3.15	1.278	0.630
Difficulty in submitting assignments on to Blackboard	30.3	36.4	18.2	9.1	6.1	2.24	1.173	0.448

*(b) Students’ satisfaction with the instructors’ performance on online teaching*

Table 4 shows the percentage, mean, standard deviation, and RII for each factor considered in this section of the survey. Overall, there is the highest level of satisfaction with the instructor’s engagement with students in discussions (RII = 0.716), encouraging students to ask questions and support to minimize difficulties (RII = 0.716). Conversely, adjustments and modifications of assignments received the least satisfaction level from the students. About 51.5% of the respondents are very or extremely satisfied with how the instructors encouraged them to ask questions, seek clarifications, and engage in discussions during online lectures. However, 33.4% were not satisfied or slightly satisfied with opportunities to resubmit assignments with improvements. Another 27.7% were not satisfied or slightly satisfied with timely feedback on their studies and assessments by instructors.

**TABLE 4:** Students’ opinions about instructors’ performance on online teaching.

Factors	Percentage (%)					Mean	SD	RII
	Not satisfied	Slightly satisfied	Moderately satisfied	Very satisfied	Extremely satisfied			
Explanations given by instructors about new adaptations for the courses	9.1	15.2	39.4	33.3	3.0	3.06	0.998	0.612
Modes of communication used for announcement and updates	0	15.2	39.4	33.3	12.1	3.42	0.902	0.684
Adjustments and modifications in the types of assignment accordingly	9.1	21.2	36.4	27.3	6.1	3.00	1.061	0.600
Adjustments and modifications in the types of assessment (quizzes/exams) accordingly	9.1	15.2	30.3	36.4	9.1	3.21	1.111	0.642
Providing flexibility in assignment deadlines	12.1	9.1	27.3	30.3	21.2	3.39	1.273	0.678
Providing opportunities to resubmit assignments with improvements	15.2	18.2	21.2	36.4	9.1	3.06	1.248	0.612
Providing timely feedback on assignments and assessments	18.2	9.1	27.3	24.2	21.2	3.21	1.386	0.642
Availability of instructors to discuss any difficulties or problems faced	6.1	12.1	39.4	21.2	21.2	3.39	1.144	0.678
Accommodation and support to students against any difficulties or problems faced	0	15.2	33.3	36.4	15.2	3.52	0.939	0.704
Encouraging students to ask questions and seek clarifications during or at the end of each online lecture	6.1	12.1	30.3	27.3	24.2	3.52	1.176	0.704
Encouraging students to engage in discussions during or after the lectures	0	15.2	33.3	30.3	21.2	3.58	1.001	0.716
Concluding the course according to the course learning outcomes	12.2	15.2	33.3	24.2	15.2	3.15	1.228	0.630

*(c) Students’ participation in online learning*

**FIGURE 2:**
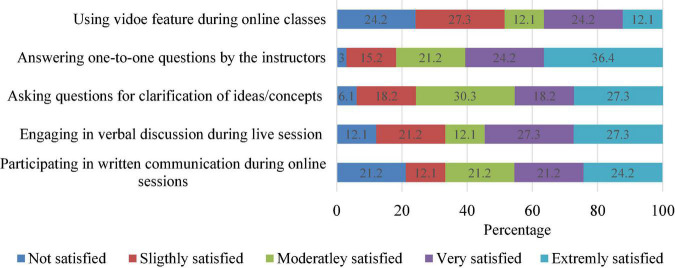
Students’ satisfaction with participation during online learning.

Figure 2 presents the results on participants’ attitude to engage with interactive functions of the live e-learning session. This study finds a varying degree of satisfaction for each question asked. For example, about 36.4% of the respondents see the platform as extremely satisfying for answering questions by the instructors. In addition, engaging in verbal discussions was also considered extremely satisfactory (27.3%) among the participants. In comparison, 24.2% said they were not satisfied using the live video chat to get the attention of the instructor and peers during the online session, and another 21.2% indicated dissatisfaction with the platform for written communications.

**FIGURE 3:**
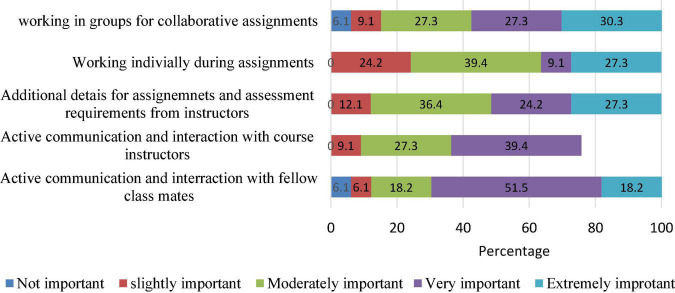
Students’ opinions about varied forms of interaction with peers and instructors.

Figure 3 presents students’ assessment of the importance of various forms of interactions with other students and instructors using the online learning platform. Close to one-third of the respondents (30.3%) considered the medium extremely important for working in group assignments. Using the platform for working individually or obtaining additional details for assignments are interactions deemed very important by 27.3% of the respondents. However, 24.2% of them considered the medium slightly important for working on assignments individually.

*(d) Students’ preference for different modes of communication*

**FIGURE 4:**
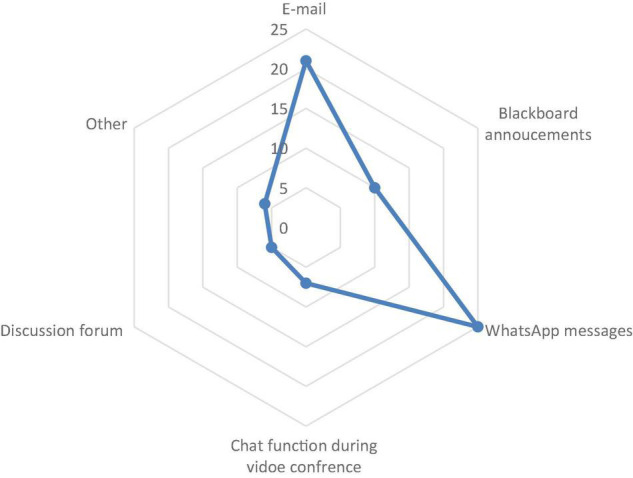
Students’ preference of different modes of communication.

Figure 4 reveals that, overall, participants expressed a greater (27%) preference for WhatsApp messaging, followed by email correspondence (22%) and Blackboard announcements (10%). The discussion forum is their least preferred mode of communication. This result is not surprising given the country’s high rate of internet penetration and the use of social media (Almulhim and Abubakar, 2021).

### Results From Focus Group Discussion

The findings of the FGD were categorized into the resulting five themes discussed below. Quotations of students’ expressions exemplify the common themes originating from the discussion sessions. The results are then discussed within the context of the existing body of knowledge.

*(a) Difficulty in concentrating during lectures*

Students frequently complained about not being able to focus on the online lectures and the difficulty in concentrating. Losing attention during online classes is often because of distractions from other activities, such as chatting with friends and surfing the web, happening simultaneously (Govindarajan and Srivastava, 2020). As a result, students often found the online sessions not engaging and ‘boring’ and struggled to remain focused throughout the lectures. One student stated, “*I could not focus on the lectures*…*there was (frequent) noise in the background.*” Another source of lack of interest during lectures is having “*too much information and text*.” Distractions by family members at home also contribute to loss of focus and difficulty concentrating on online education.

Regarding the difficulty in concentrating during online lectures, students frequently mentioned not being able to focus or concentrate. This notion corroborates Ogunleye’s (2010) study that concluded that online education might have little to offer to arouse students’ interest. Similar findings were also reported by Mukhtar et al. (2020), who found that students considered limited attention span a disadvantage of online education. Moreover, technical issues, weak connections, and delays in starting the online classes may further cause discontentment and frustration among online learners (Mackey and Freyberg, 2010).

*(b) Internet connectivity and technical issues*

Poor signals from the telecommunication network and slow internet connection are some technical issues that undermine the transition to online education. A few students said they frequently experienced problems accessing the links to the QuestionMark exams because of slow or broken connectivity during online learning. For example, one of the students had a technical issue and reported: “*My link did not work. I tried from the phone and also from the laptop, but it did not work*” (referring to QuestionMark Quiz, the issue was resolved later). Another student had a connectivity issue because he lived in a remote area. In addition, the group faced occasional problems with assignment submissions through the Blackboard, mostly because of low internet bandwidth or connectivity issues. For example, a student who tried resubmitting an improved assignment remarked, “*the system is not allowing me to resubmit the assignment*,” to which the technical problems persisted even after the instructors deleted the earlier submission and allowed for resubmission and had to accept email submissions instead.

This issue of interrupted Internet connectivity and software technical issues is consistent with that of the study by Almulhim et al. (2020), who found that problems with the Internet connection were a major source of discontentment among medical students in Saudi Arabia. Thus, the high demand for internet services during online learning is a likely contributor to technical problems encountered.

*(c) Increased workload*

As a result of a sudden transition to the online mode of learning, there was a common feeling among the students of being overburdened with several tasks from other courses. They also indicated finding it very difficult to handle all the assigned tasks simultaneously. For example, one student complained that: “*too many instructors are giving us work, and I find it not easy (to handle)*.” This concern by the students about increased workload has also been reported in earlier studies. For example, Roby et al. (2013) reported that surveyed students mentioned more workload as one of the disadvantages of online education in their studies. However, in this case, it could just be a misconstrued perception by the students as instructors in the department confirmed that the course requirements were rather lowered instead of increased.

*(d) Difficulty with quiz/exam formats*

Another issue brought up by the students in the FGD was the lack of familiarity with the QuestionMark format and how to engage with an online quiz as they found it difficult to navigate through the platform. For instance, one student said he had trouble understanding the task format and said, “*It was difficult for me to attempt the quiz, even though I prepared very well for it*.” Another student remarked, “*I did not know how many total questions I have to attempt*… *I will prefer to solve (the quiz) with my sequence.*” Whereas one student said, he did not expect the kind of question he got in the quiz. “*I was not prepared for the last question*” (referring to a descriptive question).

In addition, several students mentioned that the time for completing online quizzes was not enough compared to paper-based quizzes before the online transition. “*The time given for the quizzes was very less than before*.” Besides, there was a feeling among the group that *“the questions should be easy*,” implying that they should either be based on multiple-choice questions (MCQ) only or the number of MCQ should be more than descriptive and analytical questions. However, students were more interested in MCQ or definitions than in descriptive and analytical questions during online quizzes and exams. This finding is consistent with the study conducted by Toufaily et al. (2018), where the students mentioned not being able to sit in front of the computer for too long as a disadvantage of online education.

*(e) The need for recorded lectures*

Another observation raised by the group was the lack of recorded lecture sessions for later use. The participants suggested recording and giving them the lectures at the end of classes instead of PDF versions. One student said: *“I want to be able to go back to the lecture and watch it again*…*not through simple (PDF) lecture.”* Also, some participants suggest that a shortened lecture session will be much easier to engage with, as summed up in the words of one of them: *“It is not possible for us to sit for 2 h in online mode. Time for lecture should be short.”*

### Instructors’ Experiences of Online Course Delivery

The findings reported in this section are brief accounts of instructors’ experiences of online course delivery during the Pandemic. The accounts are based on their observations, activity logs, and individual feedback received from students. The experiences are organized around online lecture delivery, assignments and grading, communicating with students, and technical issues.

*(a) Online lecture delivery*

For the first few lectures, the class timings were not followed as scheduled to allow the students some time to adjust to the new learning modalities. Therefore, the initial lectures were scheduled after discussing with the students about the best possible time that suits all of them (see Box 1 for example). The literature indicated that the first online teaching experience could be challenging for instructors and students because of likely confusion and ambiguity for those without prior experience (Schmidt et al., 2013). Since it was the first time the instructors delivered a course online, many new experiences and observations were made. Some were positive, and some were not very promising.

**Box 1. Flexible lecture hours to allow students to adjust to the online mode of learning.**

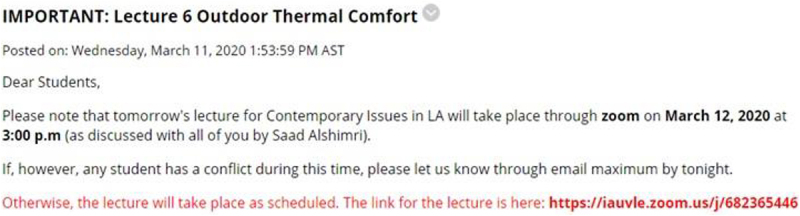

The instructors observed that most students lose interest in the lecture after the first 30 min and stop giving proper attention in the remaining lecture duration. This issue persisted throughout the course delivery, even with modifications to the lectures and shortening the duration. After some deliberation, the course instructors introduced more interactive ways, such as watching online short movies related to the course outline (see Box 2). Another strategy is asking students to fill in pre-designed templates to summarize their understanding of the topics discussed. This strategy proved effective as the students showed much interest, and submissions were made within the time limits set out for them.

**Box 2. Alternative teaching strategies to improve students’ concentration.**

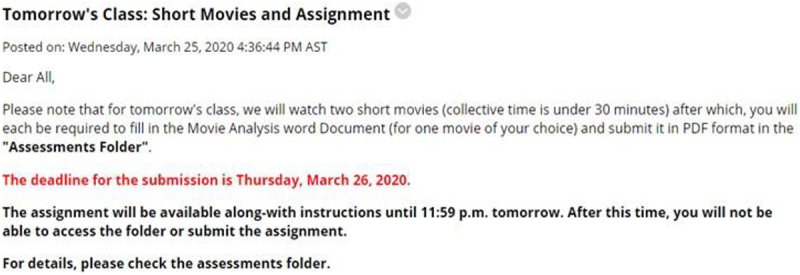

Taking attendance is another challenge faced by instructors during online lectures. The process consumes time and students feel bored. Likewise, tracking who was present is difficult and distracts the instructors from class delivery as they cannot do both simultaneously. Furthermore, some students were not interested in asking questions even when individually prompted during or after the lectures. They rather wanted the instructors to quickly end the online session in order to leave as soon as possible. As such, instructors tried to ask random questions to increase students’ attention. Nevertheless, even when called out by names, several students could not respond to the questions, implying they either physically left the session while still logged in or had technical issues. This issue is particularly concerning since some students always distract themselves from the learning atmosphere. However, the instructors did not press this issue beyond a considerable limit due to the special circumstances involved. Many students were genuinely going through difficult times and were facing countless challenges to learn online. A similar experience of inactive students’ participation in an online course during the Pandemic was reported in Hong Kong (Moorhouse, 2020). While sharing community college instructors’ experiences in online learning, Hulett (2018) notes that having higher motivation is a crucial factor in affirming students’ success.

*(b) Course assignments and workload*

The students indicated some problems that prohibited them from submitting the assignments online during the first few days. One possible explanation for this is the excessive internet and e-learning system usage and accessibility issues about weak internet access. However, the concerned IT personnel solved most of these issues, and the instructors allowed resubmissions to all students (see Box 3).

**Box 3. Announcement about resubmitting the assignments after technical issues were reported.**

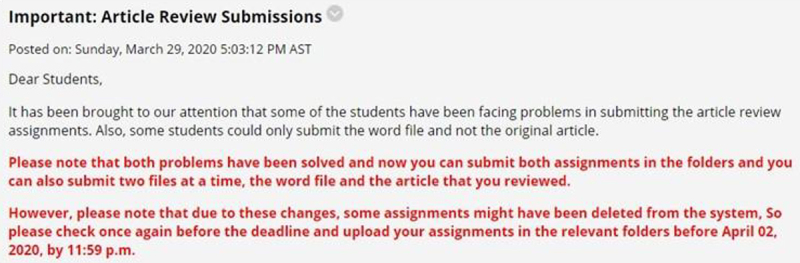

Grading the assignments was another challenge faced by the instructors. They always use the approved grading rubrics, compare the quality of work among other students, and peer-review their assessments to ensure fairness. Also, grading is easier and faster in paper-based assessments as the instructors could shuffle the pages to revisit other questions than *via* the *Blackboard* easily. Nonetheless, the instructors found that reconciling grades is best done through *Zoom* meetings, although it required considerable time.

Another challenge was receiving and managing assignments through emails. After some time, it became quite difficult to differentiate between subsequent and current assignments, and some files were even corrupted. Therefore, the instructors changed the medium of submissions to online, gave detailed written feedback, and allowed resubmissions to improve their performance (see Box 4). However, despite the opportunity, very few students improved their assignments.

**Box 4. Announcement for resubmitting assignments after improvement.**

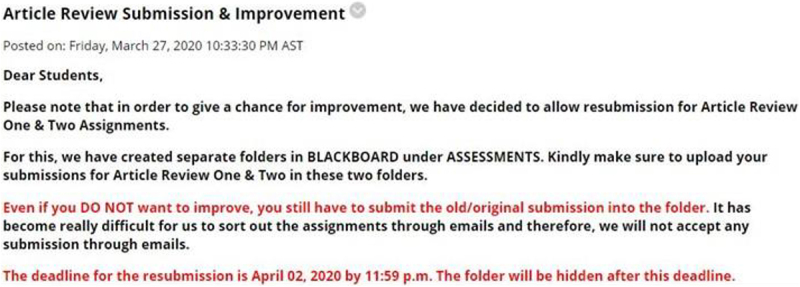

*(c) Online quizzes and examinations*

The students were also given details about the online quizzes and links for related lectures, similar to assignment instructions. The instructors realized the need to inform students beforehand about what to do in case of technical difficulties during online quizzes/examinations. Furthermore, due to anticipated technical issues in the remaining quizzes/exams, the students were informed about the proper course of action in case of any technical problems (see Box 5). In addition to guidelines for facing any technical difficulties, the instructors also sent instructions about the timing, duration, and related details through announcements and emails (see Box 6). Also, the same detailed information was sent to the students in the *WhatsApp* group through the class representative.

**Box 5. Instructions about the course of action in case of technical difficulties during online quizzes/exams.**

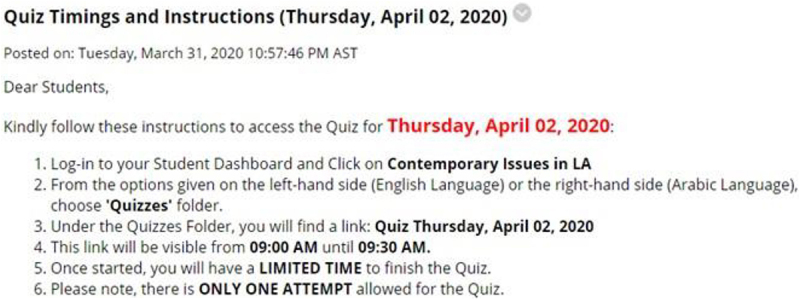

**Box 6. Announcing online quiz timings and instructions.**

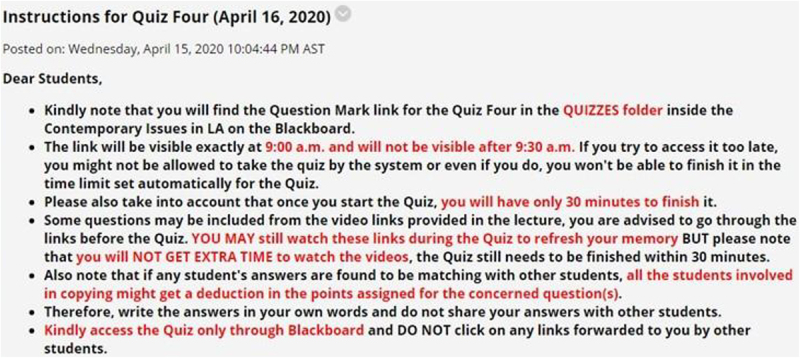

However, some issues persisted, such as students forwarding inaccessible links to other students, which did not work as each student must access the online assessment links through his account on the *Blackboard* and not through forwarded links. Moreover, it was found that having the whole quiz as MCQ is not a good idea since it increases the chances of cheating, and there is no way of making sure it does not happen in an online quiz or exam. The instructors found considerable evidence that some students had copied the exact answers from one another. Therefore, the students were informed that the subsequent quizzes would contain analytic or descriptive questions in addition to MCQ. Moreover, the instructors resorted to more detailed instructions, and the students were also repeatedly advised not to access online assessments through forwarded links (see Box 7).

**Box 7. Added details and instructions for online quizzes/examinations.**

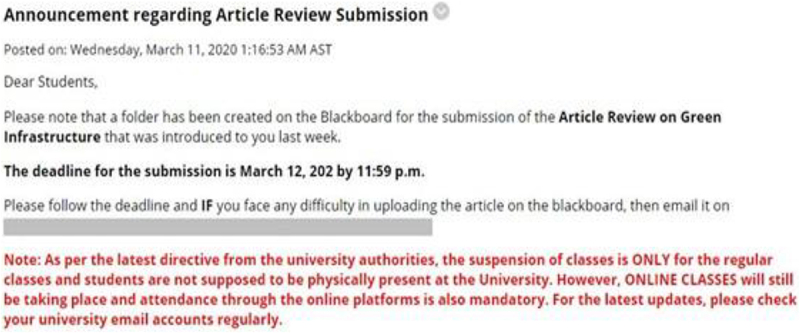

Making, publishing, and marking quizzes *via* the *QuestionMark* platform consumed more than three times the time for conventional paper-based assessment. This process can be particularly challenging because of the need to reconcile grades to avoid any bias or mistakes. The Pandemic has also increased the workload for teachers who had to rapidly transform courses and assessments designed for face-to-face to online education. For instructors, it means spending more time in logistics than improving the quality of teaching and learning (Dumford and Miller, 2018).

*(d) Communication and feedback*

Upon the suspension of face-to-face classes, the students were advised to check their emails for official announcements related to the course. The students were not interested in having a video call and always had their cameras off. Likewise, most students had their mics muted as well. This part was particularly peculiar for the instructors as there was no way of finding out how well the students were receiving the information. Despite multiple reassurances to contact the instructors, the students still seemed reluctant to contact the instructors to discuss any problems or issues related to the course. A similar concept has previously been mentioned by Serwatka (2002) that the students might show reluctance in asking for help if they face problems in online courses.

The feedback on assignments is crucial for students and helps them ‘validate’ their work (Serwatka, 2002). Before COVID-19, the course instructors gave face-to-face feedback to the students, allowing them time to read the comments first and then come to the instructors for any additional explanations. However, during the online phase, the instructors had to give written feedback against the individual submissions on the Blackboard. Therefore, it was largely undetermined whether the students seriously considered this feedback or not. The students were routinely reminded to contact any instructors through emails, WhatsApp, or even phone calls when necessary.

*(e) Technical issues*

Technical issues are fewer among the instructors than students, therefore, affected their learning activities, online assessments, and assignment submission processes. Due to the overburdened learning systems, which were not ready for such situations, the students frequently complained about not being able to access the Blackboard or, in other cases, unable to submit assignments properly. The instructors believe that this is one of the reasons why the students were dissatisfied and less motivated with the online experience.

Table 5 below summarizes the challenges faced by both students and instructors during the transition to online teaching and learning. The students experienced most difficulties related to low motivation, disruptive home working environment, and technical issues. These challenges are likely because students are overwhelmed by circumstances warranted by the lockdowns. For instance, students that mostly lived on campus within the academic setting (hostels, libraries, classrooms, etc.) before the Pandemic but were sharing their living spaces and learning resources with family members during the lockdowns. As such, concentrating on academic endeavors can be challenging due to distractions, an unsuitable learning environment, and depression due to rising COVID-19 mortality and morbidity cases.

**TABLE 5:** Summary of key challenges identified by the students and instructors.

Categories	Challenges faced by students	Challenges faced by instructors
Online lecture delivery	• Lack of motivation and concentration during lectures • Lack of proper (home) environment for online learning	• Recording attendance • Keeping students concentrated • Completing the lectures in a short time • Knowing if the students understood the lecture contents
Course assignments	• Feeling overburdened due to other coursework • Lack of interest in resubmitting assignments	• Restructuring assignments to keep the course learning outcomes the same. • Reconciling grades between the instructors
Online quizzes/ examinations	• Unfamiliarity with quiz/exam formats • Internet connectivity: slow connectivity and lack of signals •Lack of time for attempting the quiz/exams	• Preparing, publishing, and administering online quizzes/examinations. • Comparing student work and reconciling grades. • Controlling cheating during the quizzes/examinations
Communication and feedback	• Lack of camaraderie among peers • Lack of direct contact for feedback with instructor	• Motivating students to ask questions • Proper registration of feedback
Technical issues	•Internet connectivity and accessibility issues • (Occasional) difficulties in submitting assignments	• Accessing online assessment folders due to overburdened virtual learning systems. • Marking assignments during fluctuating internet accessibility

On the other hand, the instructors were challenged by managing students’ participation in online course delivery, technical issues, and creating, posting, and grading quizzes and examinations. The instructors strongly felt a lack of motivation and positive spirit among the students, which indicates that the students were largely dissatisfied with the online experience in the initial phase. These experiences have important implications for improving online course delivery in universities. The next section concludes with the lessons learned from the present study and recommends some practices toward fostering online education.

## Conclusion and Recommendations

The COVID-19 Pandemic has caused several changes in teaching and learning, negatively impacting educational institutions’ stakeholders, including students, faculty, and administrators. Faculty members and students worldwide faced difficulties in shifting to online education during the Pandemic. This study researched the challenges regarding the sudden transition from face-to-face to the an online mode of instruction and shared the experiences of students and instructors of a University in Saudi Arabia regarding the modifications to adaptations that ensued. The University made and supported timely adaptations by procuring relevant education software and programs like Zoom and QuestionMark and conducting extensive training workshops for all the faculty members to help them adapt to the sudden shift to the online mode of instruction. These training sessions have enhanced the instructors’ pedagogical and technical skills.

The students most experienced difficulties were low motivation, disruptive home working environment, and ICT issues. Conversely, the instructors faced challenges of maintaining students’ participation in online teaching, assessing students through quizzes and examinations, and technical issues. The present study also described how the instructors took various adaptation measures in the existing pedagogical framework to transition to online learning. The current research was limited to a sample of 33 students and three instructors from the College of Architecture and Planning and might not be generalized to other disciplines. However, the experiential learning experience shared in the present study makes several noteworthy contributions that can guide some faculty members to validate their findings from the course adaptations they made during the Pandemic. Furthermore, the study may give some positive insights about future planning considerations in case the Pandemic forces longer university closures or if a similar situation arises in the future. The following are the key recommendations of the present study.

### Online Lecture Delivery

• Instructors should carefully plan any course adaptations to achieve effective online course delivery during the current transition and future similar cases. They should dedicate adequate time to prepare and adapt to the learning environment to guarantee student-centered learning and a rich learning experience. Lecture duration should be shortened to avoid low interest among students and increase students’ satisfaction in online courses (Jackson et al., 2010). In addition, short videos from educational websites or *YouTube* can make lectures more interesting. Instructors could also utilize open-source educational resources to cater to the limited preparation time (Huang et al., 2020).
• If increasing student interaction is difficult, instructors could include bonus grades for active discussions and class participation to motivate students. Short oral quizzes can be introduced at the end of each lecture to give students an incentive to be attentive. Because many students are concerned with a meaningful learning experience (Brocato et al., 2015), they can participate in an active, interactive learning environment (Driscoll et al., 2012).
• Course instructors should develop norms for discussions and synchronous interaction in advance and might use examples and rubrics to explain to the students’ (Mayes et al., 2011). Discussion forums are an important part of online learning, and instructors should promote those outside class hours (Hulett, 2018).
• Students should also be prompted to ask questions during or after lectures. They should be mandated to post questions through the chat function, while the instructor answers them during or after classes. Encouraging students to explore more on the topic and giving presentations might also be helpful in this regard (Atreya and Acharya, 2020).

### Course Assignments and Workload

• Institutional and faculty supports are important for students to adapt to online learning modalities, especially under lockdown circumstances. Students should also be acquainted with the unique demands of online environments such as increased cognitive discipline, independence, and personal responsibility (Mayes et al., 2011) and intrinsic motivation, maturity, good time management, and active participation in in-class activities (Hulett, 2018).
• Course instructors should consider that students might be overburdened and exhausted due to the workload from other courses. Moreover, the circumstances related to the Pandemic itself have also been reported to increase stress among university students in Saudi Arabia (AlAteeq et al., 2020).
• The instructors could manage initial, pre-submission evaluations and give feedback before grading the assignments to enhance student performance. This strategy might encourage students to improve the assignments before submitting them for final assessments.
• Wherever possible, the instructors should not impose strict submission deadlines and allow some flexibility to the learners.
• Instructors should devise innovative methods for introducing group work and course learning outcomes that require collaboration and teamwork because during the Pandemic students could not go outside their homes, and face-to-face interaction was hampered.
• One way, for example, could be for the groups to meet separately, following the guidelines and SOPs for safe distancing. If site visits or practical work are not possible, similar activities should be introduced based on the same learning outcomes. Serwatka (2002); Stallings (2002) propose that in online teaching settings, the criteria for assessment should be revised to assess student mastery.

### Online Quizzes/Examinations

• Assignments and homework should supplement online assessments that are challenging, especially when courses involve practical skills or technical competencies. These alternative assessment methods should be based on relevant rubrics (Osman, 2020). Students find online exam formats unconventional and difficult since face-to-face education is the mainstream mode of teaching and learning before the Pandemic.
• Students should be familiar with the format and the typology of questions and the time allowed beforehand to prepare adequately. This task could be done by asking students to take mock examinations to become familiar with the format and sharing detailed, step-by-step instructions.
• Although students might prefer the easier way of having only MCQ in quizzes and exams, higher-order, cognitive skills-based questions are also recommended to ensure consistent course learning outcomes.
• To avoid cheating, instructors could make a large question bank and choose options such as shuffling the question sequence and selecting random questions for each student. The same method has previously been endorsed by Serwatka (2002).
• Making online MCQ based on the same cognitive level and course learning outcomes as paper-based quizzes/exams needs extensive revisions. Therefore, instructors should prepare such questions way ahead of the scheduled quiz or exam date or preferably at the beginning of the course.

### Communication and Feedback

• The key to effective communication between the students and instructors is finding different avenues to connect with them (e.g., emails, phones, virtual meetings, social media) and removing barriers to communication (Schmidt et al., 2013).

• Introducing online office hours could help students feel free to contact instructors during these hours. This recommendation is important because students might feel hesitant to interrupt the instructors in their private time or decide when to contact them to discuss issues or seek any clarifications on learning (Serwatka, 2002).
• Students should be given immediate and constructive feedback on course tasks and discussions (Mayes et al., 2011). However, as reconciling grades and comparing the student work could be time-consuming, instructors should allow sufficient time between assignment deadlines and assessment schedules to avoid overburdening themselves and providing prompt feedback to the students.
• Future online courses should include a student-instructor ‘camaraderie’ (friendship), through avenues such as ‘coffee shop’ or ‘teacher’s lounge’ to promote interaction, support students and develop trust (Mayes et al., 2011).

### Technical Issues

Educational institutions should provide students and instructors with prompt ICT support whenever needed. Technical support facilitates online education and teachers’ responsibilities (Roby et al., 2013). Having technical issues related to the e-learning system could impede students’ progress and their perceptions of its usefulness (Almaiah et al., 2020), exacerbate inequality in online education and intensify the digital divide among students (Govindarajan and Srivastava, 2020). Instructors in online environments need to put more effort into helping students resolve technical issues, especially under the circumstances such as the current Pandemic.

## Data Availability Statement

The original contributions presented in the study are included in the article/supplementary material, further inquiries can be directed to the corresponding author/s.

## Ethics Statement

Ethical review and approval was not required for the study on human participants in accordance with the local legislation and institutional requirements. Written informed consent for participation was not required for this study in accordance with the national legislation and the institutional requirements.

## Author Contributions

BA and SI conceptualized the study, collected the data, and wrote the initial draft. IA analyzed and interpreted the quantitative data, revised and edited the resubmitted manuscript, and addressed reviewers’ comments. All authors have approved the final version of the manuscript and agreed to be accountable for all aspects of the work.

## Conflict of Interest

The authors declare that the research was conducted in the absence of any commercial or financial relationships that could be construed as a potential conflict of interest.

## Publisher’s Note

All claims expressed in this article are solely those of the authors and do not necessarily represent those of their affiliated organizations, or those of the publisher, the editors and the reviewers. Any product that may be evaluated in this article, or claim that may be made by its manufacturer, is not guaranteed or endorsed by the publisher.

## References

[B1] Abubakar I. R. Aina Y. A. Alshuwaikhat H. M. (2020). Sustainable development at Saudi Arabian universities: an overview of institutional frameworks. *Sustainability* 12:8008. 10.3390/su12198008

[B2] Alali A. S. Xanthidis D. (2014). An exploratory study of eLearning challenges and opportunities in the GCC. *2014 World Symp. Comput. Appl. Res.* 2014 1–6. 10.1109/WSCAR.2014.6916785

[B3] Alarabiya News (2020). *Saudi Arabia Suspends All Schools Until Further Notice Amid Coronavirus Concerns.* Dubai: Alarabiya Media.

[B4] AlAteeq D. A. Aljhani S. AlEesa D. (2020). Perceived stress among students in virtual classrooms during the COVID-19 outbreak in KSA. *J. Taibah Univ. Med. Sci.* 15 398–403. 10.1016/j.jtumed.2020.07.004 32837508 PMC7395822

[B5] Aldiab A. Chowdhury H. Kootsookos A. Alam F. (2017). Prospect of eLearning in higher education sectors of Saudi Arabia: a review. *Energy Procedia* 110 574–580. 10.1016/j.egypro.2017.03.187

[B6] Almaghaslah D. Alsayari A. (2020). The effects of the 2019 Novel Coronavirus Disease (COVID-19) outbreak on academic staff members: a case study of a pharmacy school in Saudi Arabia. *Risk Manag. Healthc. Policy* 13 795–802. 10.2147/RMHP.S260918 32765134 PMC7369415

[B7] Almaiah M. A. Al-Khasawneh A. Althunibat A. (2020). Exploring the critical challenges and factors influencing the E-learning system usage during COVID-19 pandemic. *Educ. Inform. Technol.* 25 5261–5280. 10.1007/s10639-020-10219-y 32837229 PMC7243735

[B8] Almulhim A. I. Abubakar I. R. (2021). Understanding public environmental awareness and attitudes toward circular economy transition in Saudi Arabia. *Sustainability* 13:10157. 10.3390/su131810157

[B9] Almulhim A. Y. Almulhim S. A. Almulhim A. A. Khan A. S. (2020). The impact of e-learning modalities on medical students in KSA during the COVID-19 pandemic. *J. Taibah Univ. Med. Sci.* 15 437–438. 10.1016/j.jtumed.2020.08.001 33132815 PMC7564936

[B10] Atreya A. Acharya J. (2020). Distant virtual medical education during COVID-19: half a loaf of bread. *Clin. Teach.* 17 418–419. 10.1111/tct.13185 32558269 PMC7323172

[B11] Breen R. L. (2006). A practical guide to focus-group research. *J. Geogr. High. Educ.* 30 463–475. 10.1080/03098260600927575

[B12] Brocato B. R. Bonanno A. Ulbig S. (2015). Student perceptions and instructional evaluations: a multivariate analysis of online and face-to-face classroom settings. *Educ. Inform. Technol.* 20 37–55. 10.1007/s10639-013-9268-6

[B13] CNN Arabic (2020). *Saudi Arabia Records The First Case Of Coronavirus Infection Of A Citizen Who Was In Iran Without Disclosing This.* Dubai Media City: CNN Arabic.

[B14] Driscoll A. Jicha K. Hunt A. N. Tichavsky L. Thompson G. (2012). Can online courses deliver in-class results? *Teach. Sociol.* 40 312–331. 10.1177/0092055x12446624

[B15] Dumford A. D. Miller A. L. (2018). Online learning in higher education: exploring advantages and disadvantages for engagement. *J. Comput. High. Educ.* 30 452–465. 10.1007/s12528-018-9179-z

[B16] Govindarajan V. Srivastava A. (2020). *What the Shift to Virtual Learning could mean for the Future of Higher Ed.* Brighton: Harvard Business Review.

[B17] Herrington A. Herrington J. Oliver R. Stoney S. Willis J. (2001). “Quality guidelines for online courses: the development of an instrument to audit online units,” in *Proceedings of 18th Conference of the Australasian Society for Computers in Learning in Tertiary Education.* (Melbourne: Biomedical Multimedia), 263–270.

[B18] Huang R. Tlili A. Chang T.-W. Zhang X. Nascimbeni F. Burgos D. (2020). Disrupted classes, undisrupted learning during COVID-19 outbreak in China: application of open educational practices and resources. *Smart Learn. Environ.* 7 1–15. 10.1186/s40561-020-00125-8 PMC7336082 40476981

[B19] Hulett M. T. (2018). *Online Teaching Strategies & Best Practices.* Ph.D.thesis. Long Beach: California State University.

[B20] Iqbal S. A. Tayyab N. (2021). Letter to Editor. *J. Loss Trauma* 26 97–100. 10.1080/15325024.2020.1806575

[B21] Jackson L. C. Jones S. J. Rodriguez R. C. (2010). Faculty actions that result in student satisfaction in online courses. *J. Asynchronous Learn. Netw.* 14 78–96. 10.24059/olj.v14i4.129 33692645

[B22] Johnson N. Veletsianos G. Seaman J. (2020). U.S. faculty and administrators’ experiences and approaches in the early weeks of the COVID-19 pandemic. *Online Learn.* 24 6–21. 10.24059/olj.v24i2.2285 33692645

[B23] Jones S. H. (2014). Benefits and challenges of online education for clinical social work: three examples. *Clin. Soc. Work J.* 43 225–235. 10.1007/s10615-014-0508-z

[B24] Kaden U. (2020). COVID-19 school closure-related changes to the professional life of a K–12 teacher. *Educ. Sci.* 10:165. 10.3390/educsci10060165

[B25] Khalil R. Mansour A. E. Fadda W. A. Almisnid K. Aldamegh M. Al-Nafeesah A. (2020). The sudden transition to synchronized online learning during the COVID-19 pandemic in Saudi Arabia: a qualitative study exploring medical students’ perspectives. *BMC Med. Educ.* 20:285. 10.1186/s12909-020-02208-z 32859188 PMC7453686

[B26] Lederman D. (2020). *The Shift to Remote Learning: The Human Element.” Inside Higher Ed.* Available Online at: https://www.insidehighered.com/digital-learning/article/2020/03/25/how-shift-remote-learning-might-affect-students-instructors-and [accessed July 29, 2020].

[B27] Mackey K. R. M. Freyberg D. L. (2010). The effect of social presence on affective and cognitive learning in an international engineering course taught via distance learning. *J. Eng. Educ.* 99 23–34. 10.1002/j.2168-9830.2010.tb01039.x

[B28] Marinoni G. van’t Land H. Jensen T. (2020). *The Impact of COVID-19 on Higher Education Around the World - IAU Global Survey Report.* Paris: International Association of Universities.

[B29] Mayes R. Luebeck J. Ku H.-Y. Akarasriworn C. Korkmaz Ö (2011). Themes and strategies for transformative online instruction: a review of literature and practice. *Q. Rev. Distance Educ.* 12 151–166.,221–222, 10.1177/2382120520943595 32754648 PMC7378721

[B30] Moorhouse B. L. (2020). Adaptations to a face-to-face initial teacher education course ‘forced’ online due to the COVID-19 pandemic. *J. Educ. Teach.* 46 609–611. 10.1080/02607476.2020.1755205

[B31] Mukhtar K. Javed K. Arooj M. Sethi A. (2020). Advantages, Limitations and Recommendations for online learning during COVID-19 pandemic era. *Pak. J. Med. Sci.* 36 S27–S31. 10.12669/pjms.36.COVID19-S4.2785 32582310 PMC7306967

[B32] Ogunleye A. O. (2010). Evaluating an online learning programme from students perspectives. *J. College Teach. Learn.* 7 79–90. 10.19030/tlc.v7i1.82

[B33] Osman M. E. (2020). Global impact of COVID-19 on education systems: the emergency remote teaching at Sultan Qaboos University. *J. Educ. Teach.* 46 463–471. 10.1080/02607476.2020.1802583

[B34] Rajab K. D. (2018). The effectiveness and potential of E-learning in war zones: an empirical comparison of face-to-face and online education in Saudi Arabia. *IEEE Access* 6 6783–6794. 10.1109/access.2018.2800164

[B35] Roby T. Ashe S. Singh N. Clark C. (2013). Shaping the online experience: how administrators can influence student and instructor perceptions through policy and practice. *Internet High. Educ.* 17 29–37. 10.1016/j.iheduc.2012.09.004

[B36] Schmidt S. W. Hodge E. M. Tschida C. M. (2013). How university faculty members developed their online teaching skills. *Q. Rev. Distance Educ.* 14 131–140.

[B37] Serwatka J. (2002). Improving student performance in distance learning courses. *T.H.E J.* 29 46–51.

[B38] Stallings D. (2002). Measuring success in the virtual university. *J. Acad. Libr.* 28 47–53. 10.1016/S0099-1333(01)00300-7

[B39] Stickney L. T. Bento R. F. Aggarwal A. Adlakha V. (2019). Online higher education: faculty satisfaction and its antecedents. *J. Manag. Educ.* 43 509–542. 10.1177/1052562919845022

[B40] Toufaily E. Zalan T. Lee D. (2018). What do learners value in online education. *e-J. Bus. Educ. Scholarsh. Teach.* 12 24–39.

[B41] World Health Organization [WHO] (2020). *Timeline of WHO’s Response to COVID-19.* Geneva: WHO.

[B42] Xanthidis D. Xanthidou O. K. Nicholas D. (2016). eLearning penetration, challenges and opportunities in Saudi Arabia. *Indian J. Sci. Technol.* 9 1–9. 10.17485/ijst/2016/v9i48/85900

